# Case Report: PAFAH1B1 Mutation and Posterior Band Heterotopia With Focal Temporal Lobe Epilepsy Treated by Responsive Neurostimulation

**DOI:** 10.3389/fneur.2021.779113

**Published:** 2021-11-18

**Authors:** Frank G. Gilliam, Paddy Ssentongo, Michael Sather, Yuka I. Kawasawa

**Affiliations:** ^1^Department of Neurology, University of Texas Rio Grande Valley, Edinburg, TX, United States; ^2^Department of Engineering Science and Mechanics, Penn State University, Hershey, PA, United States; ^3^Department of Public Health Sciences, Penn State University, Hershey, PA, United States; ^4^Department of Neurosurgery, Penn State University, Hershey, PA, United States; ^5^Department of Pharmacology and Biochemistry and Molecular Biology, Penn State University, Hershey, PA, United States

**Keywords:** band heterotopia, LIS1 gene, temporal lobe epilepsy, responsive neurostimulation (RNS), PAFAH1B1

## Abstract

Subcortical band heterotopia (SBH), also known as double cortex syndrome, is a malformation of cortical development caused by inherited or somatic gene variants. We present a case of a young adult with posterior SBH and electroclinical features of focal neocortical temporal lobe epilepsy. Genomic blood analysis identified a pathogenic somatic mosaicism duplication variant of the P*AFAH1B1* gene. Despite bilateral cortical MRI abnormalities, the interictal and ictal EEG findings indicated a focal epileptogenic region in the left posterior temporal region. Chronic responsive cortical neurostimulation across two four-contact depth electrodes placed 5 mm on either side of the maximal interictal spiking identified during intraoperative electrocorticography resulted in a consistent 28% reduction in duration of electrographic seizures and as well as constricted propagation. Although electrographic seizures continued, the family reported no clinical seizures and a marked improvement in resistant behaviors. This observation supports that focal neocortical neuromodulation can control clinical seizures of consistently localized origin despite genetic etiology, bilateral structural brain abnormalities, and continuation of non-propagating electrographic seizures. We propose that a secondary somatic mutation may be the cause of the focal neocortical temporal lobe epilepsy.

Subcortical band heterotopia (SBH), also known as double cortex syndrome, is a malformation of cortical development that is associated with cognitive problems and seizures (1). The most common causal mutations occur on the *DCX* gene (protein doublecortin) at Xq22.3–q23 (2). More prominent posterior heterotopia are seen on MRI in persons with a mosaic pathogenic mutation of the platelet-activating factor acetylhydrolase 1B1 [*PAFAH1B1*; also known as *LIS1* (3)] gene (4). We present a 32 year old patient with posterior SBH (Figures 1A,B) and a pathogenic somatic mosaicism duplication variant of the P*AFAH1B1* gene (c. 162dupA; p. Trp55MetfsX6; GeneDx, Inc., Gaithersburg, MD). She had a moderate static encephalopathy with limited vocabulary and fund of knowledge, but was able to express herself clearly. No family members had epilepsy or genetic disorder, supporting a *de novo* mutation. Her epilepsy was diagnosed at age 20 months. The seizures consisted of staring, unresponsiveness, and downward head turning to the left lasting up to 30 s. She remained irritable and depressed for many hours afterward. The seizures recurred at a rate of up to several per day, several days per week. Seven medications during 30 years of treatment had not controlled the focal seizures with impaired awareness. She had several generalized tonic clonic (GTC) seizures per year throughout adulthood, but these stopped after the addition of zonisamide to lamotrigine. Interictal scalp EEG showed frequent spike and slow wave complexes in the left posterior temporal region, maximal at the T5 (P7) electrode as shown in Figure 1C. Seizure onset consisted of low amplitude fast (beta) activity in same electrode.

**Figure 1:**
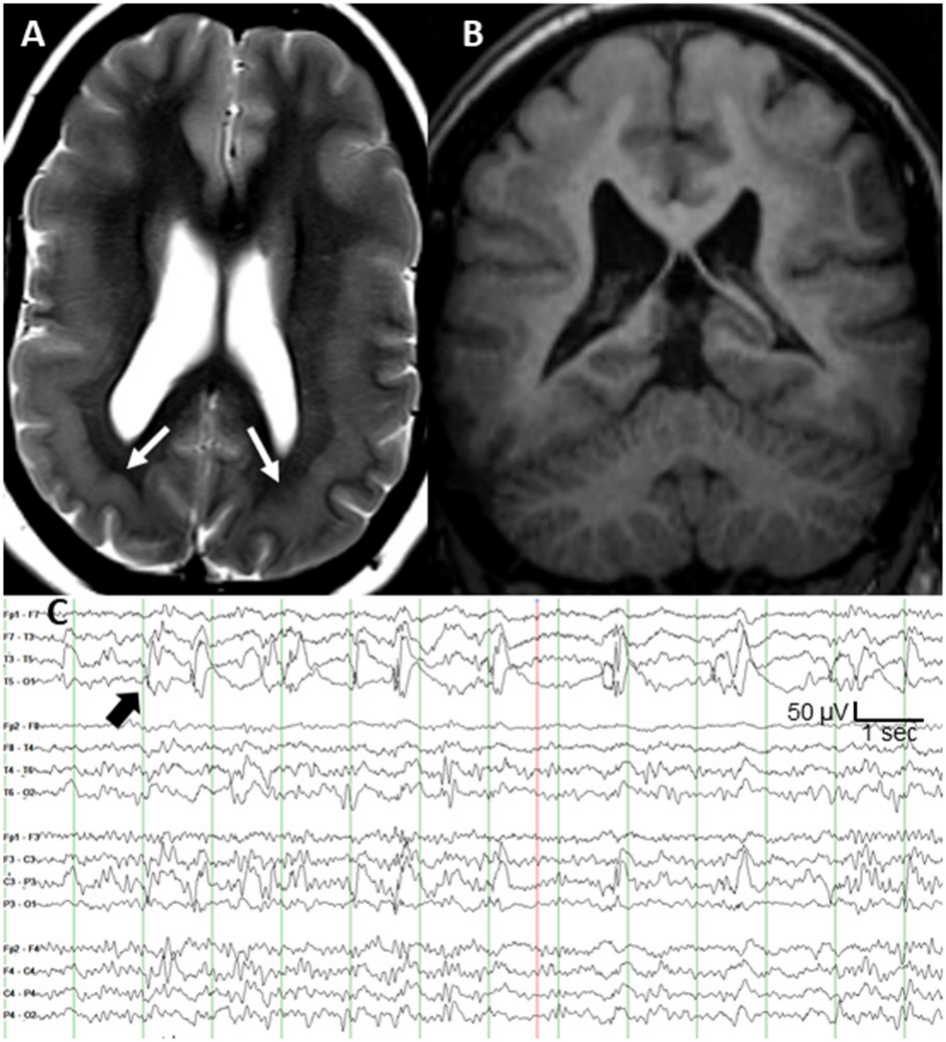
**(A)** T2-weighted axial MRI of bilateral posterior band heterotopia (white arrows), **(B)** T1-weighted coronal MRI, and **(C)** scalp interictal EEG with left posterior temporal spikes (black arrow).

After extensive discussion of treatment options, she and her family elected responsive cortical neurostimulation (Neuropace RNS®, Mountain View, CA). Following their review of the published efficacy data on vagal nerve stimulation and anterior thalamic nucleus stimulation, her parents concluded that responsive cortical stimulation was their preferred option. Also, anterior thalamic stimulation was not yet commercially available. Based on the highly focal and concordant interictal and ictal EEG findings, and frequency of the discrete interictal spikes every 1–5 s (Figure 1C), the clinical epilepsy team concluded that intraoperative electrocorticography was adequate to localize the epileptogenic region. The neurosurgeon (M.S.) performed a craniotomy large enough to allow several centimeters of cortical EEG recording around the predicted epileptogenic region based on the T5 maximal interictal EEG spikes. Intraoperative electrocorticography during 25 min of recording from a depth electrode sequentially placed at 3–5 mm increments targeting both layers of cortex across the epileptogenic region identified a single, discrete region of maximal spike amplitude. The two depth electrodes for responsive neurostimulation had four contacts each at 3.5 mm intercontact spacing, and were placed 5 mm on either side of the maximal spike negativity identified during electrocorticography. The depth of each electrode was calculated based on MRI measurements to allow two contacts in each layer of the double cortex.

Following 1 month of baseline observation, stimulation intensity was gradually increased by 0.5–1 mA at 2- to 4-week intervals. The final stimulation pathway was 5 mA across anodal contacts on one electrode to cathodal contacts on the other (charge density 2.5 μC/Cm^2^, pulse width 160 μS, duration 100 ms, and frequency 200 Hz). Her family reported a gradual decline in the intensity and duration of the clinical seizures, until they no longer observed any behaviors suspicious for seizures. The family emphasized cessation of postictal irritability and depression. She also began participating in family activities such as cooking and singing. This clinical improvement was associated with decreased electrographic seizure duration from a mean of 25–18 s (median 23 and 17 s; Wilcoxon two-sample test *p*-value <0.001) and less propagation, as shown in Figures 2A,B. Her electrographic seizure rate did not decrease. No electrographic seizures over 19 s, and no clinical seizures, were observed for a follow-up period of 18 months. No changes were made to the doses of lamotrigine and zonisamide following RNS implantation.

**Figure 2:**
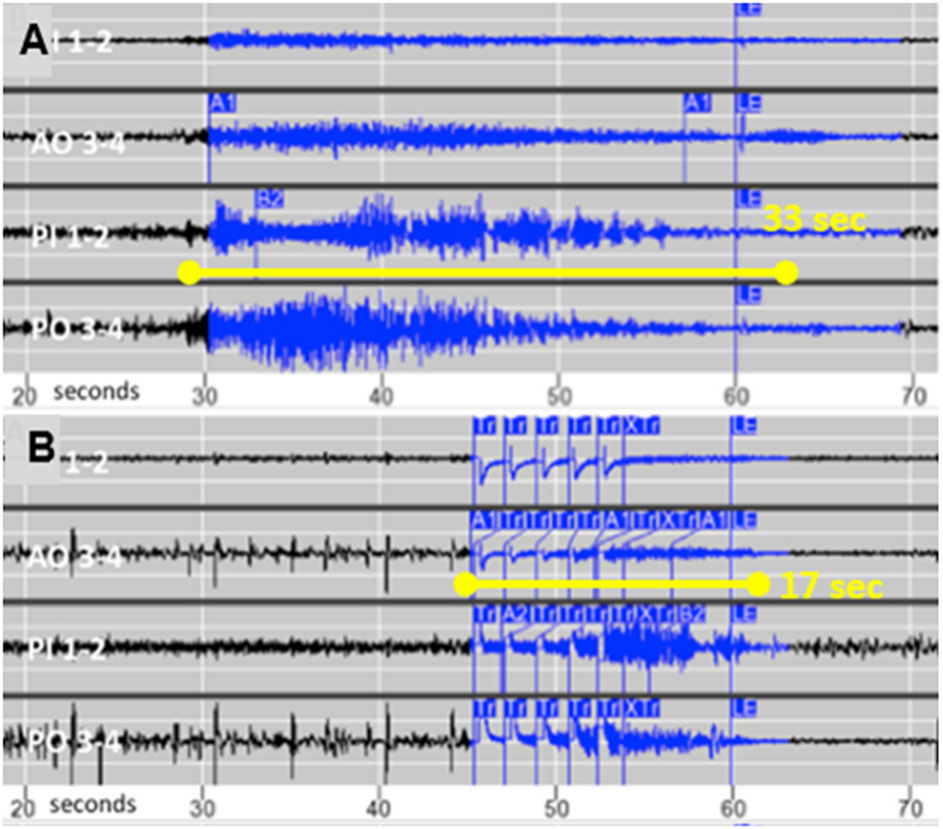
**(A)** Typical seizure recorded from chronic intracortical electrodes prior to activating stimulation and **(B)** The typical shortened seizure with less propagation following optimization of stimulation parameters.

Focal resection was reported to improve three of eight patients with SBH and evidence of a focal epileptogenic region, but the authors concluded that their results did not support the use of focal surgical resection (5). To our knowledge this is the first reported case of SBH to receive responsive focal neurostimulation, which resulted in cessation of clinical seizures as the mean duration of electrographic seizures decreased by 28%. Similar to tuberous sclerosis complex with a genetic etiology and multiple tubers, SBH patients may have consistently focal onset that responds to focal treatment (6). We speculate that focal epilepsy in SBH patients may be due to “second hit” somatic mutations, analogous to the formation of tubers in tuberous sclerosis (7). Our case also supports proof of principle that shortening seizure duration and propagation with responsive neurostimulation can eliminate clinical seizures.

## Data Availability Statement

The original contributions presented in the study are included in the article/supplementary material, further inquiries can be directed to the corresponding author/s.

## Ethics Statement

Ethical review and approval was not required for the study on human participants in accordance with the local legislation and institutional requirements. Written informed consent for participation was not required for this study in accordance with the national legislation and the institutional requirements.

## Author Contributions

All authors listed have made a substantial, direct and intellectual contribution to the work, and approved it for publication.

## Conflict of Interest

The authors declare that the research was conducted in the absence of any commercial or financial relationships that could be construed as a potential conflict of interest.

## Publisher's Note

All claims expressed in this article are solely those of the authors and do not necessarily represent those of their affiliated organizations, or those of the publisher, the editors and the reviewers. Any product that may be evaluated in this article, or claim that may be made by its manufacturer, is not guaranteed or endorsed by the publisher.
